# Exploring Distribution and Evolution of *Pi-ta* Haplotypes in Rice Landraces across Different Rice Cultivation Regions in Yunnan

**DOI:** 10.3390/genes15101325

**Published:** 2024-10-15

**Authors:** Hengming Luo, Lin Lu, Qun Wang, Zhixiang Guo, Lina Liu, Chi He, Junyi Shi, Chao Dong, Qiaoping Ma, Jinbin Li

**Affiliations:** 1 Yunnan Key Laboratory of Green Prevention and Control of Agricultural Transboundary Pests, The Ministry of Agriculture and Rural Affairs International Joint Research Center for Agriculture, Agricultural Environment and Resource Research Institute, Yunnan Academy of Agricultural Sciences, Kunming 650205, China; lhm2670150914@163.com (H.L.); qunwang70@163.com (Q.W.);; 2 Flower Research Institute, Yunnan Academy of Agricultural Sciences, Kunming 650205, China; lu_lin2005@sina.com; 3 Biotechnology and Germplasm Resources Institute, Yunnan Academy of Agricultural Sciences, Yunnan Seed Laboratory, Yunnan Provincial Key Lab of Agricultural Biotechnology, Key Laboratory of Southwestern Crop Gene Resources and Germplasm Innovation, Ministry of Agriculture/Scientific Observation Station for Rice Germplasm Resources of Yunnan, Ministry of Agriculture and Rural Affairs, Kunming 650205, China; cchaodong@126.com; 4 Honghe Academy of Agricultural Sciences, Mengzi 661100, China

**Keywords:** *Pi-ta* gene, haplotypes, rice landraces, distribution, evolution

## Abstract

**Highlights:**

Distribution of the *Pi-ta* gene was explored in rice landraces in different regions in Yunnan Province. A total of 385 rice landraces collected from these regions were found to carry the *Pi-ta* gene.

The Pi-ta haplotypes in rice landraces in Yunnan Province were identified and discovered to encode 12 novel Pi-ta protein variations.

The evolutionary cluster and network of the *Pi-ta* haplotypes in rice landraces in Yunnan Province were analyzed. These results suggest that the *Pi-ta* haplotypes carrying *R* alleles evolved from *S* alleles in these rice landraces.

**Abstract:**

**Background**: Rice blast, caused by *Magnaporthe oryzae*, seriously damages the yield and quality of rice worldwide. *Pi-ta* is a durable resistance gene that combats *M. oryzae* carrying *AVR-Pita1*. However, the distribution of the *Pi-ta* gene in rice germplasms in Yunnan Province has been inadequately studied. **Methods**: We analyzed the potential molecular evolution pattern of *Pi-ta* alleles by examining the diversity in the coding sequence (CDS) among rice varieties. **Results**: The results revealed that 95% of 405 rice landraces collected from different ecological regions in Yunnan Province carry *Pi-ta* alleles. We identified 17 nucleotide variation sites in the CDS regions of the *Pi-ta* gene across 385 rice landraces. These variations led to the identification of 28 *Pi-ta* haplotypes, encoding 12 novel variants. Among these, 5 *Pi-ta* haplotypes (62 rice landraces) carried *R* alleles. The evolutionary cluster and network of the *Pi-ta* haplotypes suggested that the *Pi-ta S* alleles were the ancestral alleles, which could potentially evolve into *R* variants through base substitution. **Conclusions**: This study suggests that *Pi-ta* alleles are diverse in the rice landraces in Yunnan, and the *Pi-ta* sites resistant to blast evolved from the susceptible plants of the rice landraces. These results provide the basis for breeding resistant varieties.

## 1. Introduction

Rice (*Oryza sativa* L.) is one of the most important food crops in many countries, including India, China, East Asia, Southeast Asia, and Africa, and plays a crucial role in catering to the nutritional requirements of 70% of the population in these regions [1]. This crop not only holds cultural significance but also forms the backbone of food security in these regions. There are several biological factors that influence the yield and quality of rice. Among them, fungi, bacteria, and viruses make the most significant contributions to disease-causing factors [2]. Rice blast, a devastating fungal disease caused by the pathogen *M*. *oryzae* (also known as *Pyricularia oryzae*), was initially reported in the United States in 1876 [3]. Strains of the fungus that have been isolated from *Digitaria* (e.g., crabgrass and fingergrass) have been designated as *M. grisea*. In contrast, those strains that have been isolated from rice and other hosts have been named *M. oryzae* [4]. The blast disease causes 10% to 30% of losses in rice yields, which can be enough to feed about 60 million people annually, and even no harvest under severe conditions [3,5]. In 2021, the Jeonbuk Province of Korea experienced a significant outbreak of rice blast. This region represents 27.7% of the country’s total rice cultivation area. Alarmingly, the extent of the outbreak was 23.7 times greater than that of 2019 and 2.6 times higher than 2020, respectively [6]. The average annual damage area of rice blast was about 75 million hm^2^ from 2013 to 2017 years in China [7]. Cultivating resistant varieties in the field has been demonstrated to be the most effective, economical, and sustainable way to control rice blast. The defensive mechanism in rice operates on the principles of the classical gene-for-gene theory. The resistance (*R*) gene in rice has the ability to recognize a corresponding avirulence (*AVR*) gene presented by the pathogen *M. oryzae*. Upon recognition, it triggers an immune response in the host plant and provides an immunoreaction against rice blast disease [8]. So far, more than 100 blast *R* genes have been identified in rice. Among these, 38 resistance genes have been successfully cloned and functionally verified [9,10]. For instance, certain rice lines that carry the *Pi9* gene, a broad-spectrum blast resistance gene located on chromosome 6, have demonstrated resistance to 43 different blast isolates [9]. These isolates were collected from 13 different countries. The *Pik-h* gene found in a wide range of cultivars, such as the indica cvs. Tetep and Tadukan, showed resistance to 12 different isolates of blast fungus collected from India [11,12]. Most of these resistance genes, including *Pi-ta*, *Pi-1*, *Pi25*, *Pigm*, and *Pia*, are dominant and encode a nucleotide-binding site and leucine-rich domain, [9]. While the *R* gene-mediated immune response is highly effective, once attracted, a cultivated variety with a monogenic line becomes susceptible after 3–5 years in the rice field. This susceptibility is due to the rapid variations of specific races, which result in the functional loss of the *AVR* gene. However, the *R* gene is coevolving with the *AVR* gene in nature [9]. Therefore, analysis of the distribution and variation of the *R* gene in germplasm resources is beneficial to evaluate its effectiveness in the field and discover the novel evolutionary haplotypes for breeding new rice varieties.

*Pi-ta* is a potent and durable resistance gene against the rice blast disease caused by *M. oryzae*, which contains the *AVR-Pita1* gene. The gene has been effectively deployed for the past 40 years to combat rice blast, and it continues to provide effective resistance not only in the United States but also worldwide [13]. The *Pi-ta* gene, known for its resistance properties, was first reported in the landrace rice variety of Taducan in the 1950. It was first cloned from the Yashiromochi rice cultivar against the blast fungus carrying the *AVR-Pita1* gene, a member of the *AVR-Pita* gene family [14,15]. The *Pi-ta* gene was located in the centromere of chromosome 12. Its coding region consists of 2 distinct sequences: CDS1 and CDS2. These sequences encode a predicted cytoplasmic protein, which consists of 928 amino acids. This protein is characterized by a nucleotide binding site and leucine-rich domains [14]. The Pi-ta protein can specifically recognize AVR-Pita1 effectors and trigger a series of defense responses to prevent infection by races of *M. oryzae* that carry the *AVR-Pita1* gene, which is known as effector-triggered immunity [15,16,17]. Immunoreaction mediated by *Pi-ta* must be assisted by a resistance gene *Ptr(t)* [18,19]. *Pi-ta* and *AVR-Pita1* are the earliest cloned race interactional pairs in blast *R* and *AVR* genes, and the specific interaction between them is determined only by the replacement mutation of a single amino acid in the Pi-ta protein at position 918, which serine (Ser-918) instead of alanine (Ala-918) impaired interaction and led to susceptibility [16]. The high degree of *Pi-ta* site polymorphism in cultivated rice and its relatives could be contributed to the selection pressure exerted during domestication, and a long terminal repeat retrotransposon located near the *Pi-ta* promoter was found in all resistant cultivars carrying the resistant *Pi-ta* gene [13]. The translation of *Pi-ta* haplotype sequences across different rice cultivars has revealed multiple variations. For example, there were 64 *Pi-ta* haplotypes found to encode 47 distinct Pi-ta protein variants in rice germplasm derived from 48 *Indica* rice accessions and publicized *Pi-ta* haplotype variants from 220 rice accessions [20]. Yunnan is the origin of multi-cropping agriculture, including rice [21]. However, the distribution of the *Pi-ta* gene in rice germplasms in Yunnan Province has been poorly studied. Therefore, investigating the distribution and variation of the *Pi-ta* gene in this region is crucial to enhance gene development and to improve our understanding of the interactions between the resistant gene *Pi-ta* and the avirulence gene *AVR-Pita1*.

Investigating the variation of the *R* gene in cultivated rice contributes to the evaluation of their effectiveness and persistence for the corresponding *AVR* gene in the field. However, the variation in the coding region sequence (CDS) in the *Pi-ta* gene and its corresponding protein products in rice germplasms from different regions in Yunnan Province remains unclear. Furthermore, the impact of this variation on the evolution of the *Pi-ta* gene is yet to be understood. In this study, we analyzed the variation and evolution of the coding region of *Pi-ta* in 385 rice germplasms collected from different regions in Yunnan Province. We discovered 12 novel *Pi-ta* variations, of which 5 novel Pi-ta proteins contained resistance (Ala-918) to rice blast and were derived from susceptible plants that hold the *Pi-ta* gene (Ser-918). This is the first report on the distribution and evolution of the *Pi-ta* gene rice landraces in different rice-growing regions in Yunnan Province. These findings have provided the basic material for resistant breeding against blast and enhanced our understanding of the effectiveness of *Pi-ta* in China.

## 2. Materials and Methods

### 2.1. Collection of Rice Landraces

In this study, we collected 405 samples from the different rice regions, including 48, 3, 1, 3, 46, 82, 128, and 94 samples from center, eastern, northeastern, northwestern, southeastern, southern, southwestern, and western, respectively, in the Yunnan Province of China.

### 2.2. DNA Preparation of Rice Landraces

The genome DNA of 405 samples collected from the different rice regions in Yunnan was extracted by the CTAB method. Briefly, the leaves of rice landraces (about 0.5 g) were prepared for extraction of total nucleic acids. Chloroform and isoamyl alcohol (24:1) were used to separate the total nucleic acids and proteins of these samples. Then, the total nucleic acids were precipitated with isopropyl alcohol and finally dissolved with 50 μL ddH_2_O for PCR amplification.

### 2.3. PCR Amplification and DNA Sequencing

A total of 2 coding sequences (CDS) of the *Pi-ta* gene were detected by using its 3 sets of specific primers in these samples of rice landraces (Table 1). Each PCR reaction was amplified in a total reaction volume of 25 µL containing the following components: 22 µL of 1.1× Mix Ver.2 (Qingke Biotech, Beijing, China), 1 µL (10 µM) of each primer, and 1 µL of DNA template. Reactions were performed in a C1000 thermal cycler (Bio-Rad, Hercules, CA, USA) with the following program: 1 cycle at 98 °C for 2 min for initial denaturation; 35 cycles at 98 °C for 10 s, 57 or 59 °C for 15 s, 72 °C for 30 s; and a final denaturation of 72 °C for 5 min. Each reaction was repeated twice. The size of the amplified DNA fragment was estimated using the DL 2000 DNA ladder (Qingke Biotech). The PCR products were sequenced using the same primers as above for PCR amplification. The DNA was sequenced by Qingke Life Technologies Biotechnology Co., Ltd. (Qingke, Beijing, China). The amplicons from each isolate were sequenced in 3 separate PCR replicates.

### 2.4. Data Analysis

The CDS1 and CDS2 sequences and the corresponding amino acids of the *Pi-ta* were assembled by DNASTAR v. 7.10 software (https://www.dnastar.com/, accessed on 27 June 2023). DnaSP v5.10.01 [22] was used for calculation of polymorphic sites (*π*), the number of DNA haplotypes, and the sliding window. Haplotype network analysis was performed using TCS v. 1.21 [23] (https://www.softpedia.com/get/Science-CAD/Posada-TCS.shtml, accessed on 29 April 2024). Fontaine’s method was used to calculate the diversity index of the rice blast fungus population [24]: diversity index = (1 − ∑^n^_i_ = 1*P*_i_^2^), where *P*i is the frequency of haplotype i in a population. Then, Tajima’s test of neutrality was performed using MEGA v. 5.10. A phylogenetic tree was constructed using MEGA v. 5.10 using the neighbor joining method [25].

## 3. Results

### 3.1. Distribution of Pi-ta Gene in Rice Landraces Collected from Different Regions in Yunnan Province

To analyze the distributions of the *Pi-ta* gene in the different rice regions in Yunnan Province, a total of 405 samples were collected from the center, northeastern, northwestern, southeastern, southern, southwestern, and western regions in Yunnan Province, in China. The CDS (CDS1 and CDS2) regions of *Pi-ta* were detected using *Pi-ta*-specific primers. There were 385 samples carrying both the CDS1 and CDS2 regions among the 405 samples, while 18 samples were carrying the CDS2 but not CDS1 regions, and 2 samples were carrying the CDS1 but not CDS2 regions (Table 2). To exclude possible contamination, each PCR was repeated 3 times. These results suggest that the *Pi-ta* gene is wildly distributed among rice landraces across different rice cultivation areas in Yunnan Province.

### 3.2. The Novel Haplotypes of Pi-ta Gene Discovered in Rice Landraces in Yunnan Province

**Figure 1:**
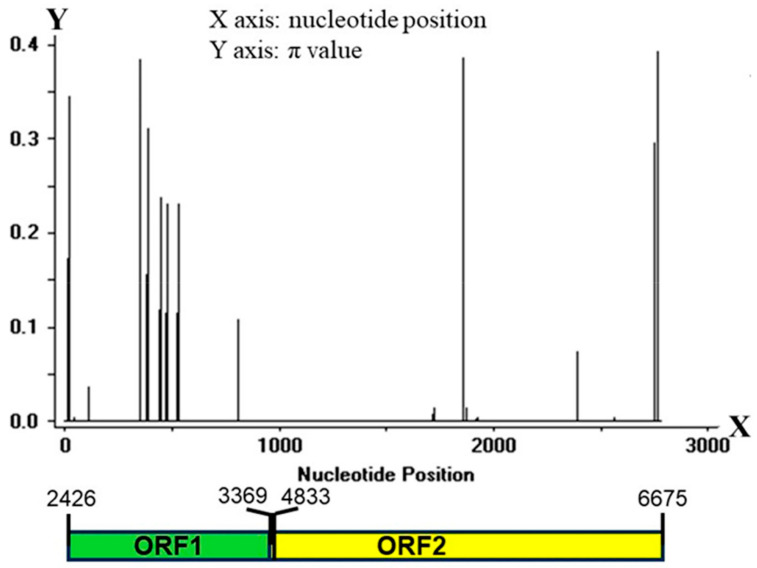
Diversification of *Pi-ta* in rice landraces in Yunnan. Distribution of variation of the *Pi-ta* alleles was analyzed using sliding window. X-axis shows the distribution of variation within the full CDS regions. Lower pane indicates the corresponding schematic presentation of the two exons of *Pi-ta*. Window length: 10; step size: 1. π value corresponds with the level of variation at each site, because it is the sum of pair-wise differences divided by the number of pairs within the population.

The *Pi-ta* locus across different rice cultivars has revealed multiple variations [20]. To investigate the diversity of the *Pi-ta* locus in the rice landraces in Yunnan Province, CDS1 and CDS2 of the *Pi-ta* gene were amplified from the 385 rice samples by using the specific primers, and the amplified PCR products were sequenced and assembled. These gene sequences were compared with the 16 reference sequences (GenBank accession: AF207842.1, EU770206.1, EU770207.1, EU770208.1, EU770209.1, EU770210.1, EU770211.1, EU770212.1, EU770213.1, EU770214.1, EU770215.1, EU770216.1, EU770217.1, EU770218.1, EU770219.1, EU770220.1), and a total of 52 mutation loci, including 35 haplotypes, were identified in the CDS of *Pi-ta* (Table S1). A total of 17 mutation loci and 28 different haplotypes (H01–H28) of the *Pi-ta* gene were found in the 385 rice landraces in Yunnan, and H01, H05, and H03 were the major haplotypes with high frequencies of 31.2%, 26.0%, and 15.1%, respectively (Table 3). The H02, H04, H6, H07, H08, H09, H10, and H11 haplotypes detected frequencies from 1.6% to 5.7%, while the remaining haplotypes were found with low frequencies (less than 1%) (Table 3). Among 28 *Pi-ta* haplotypes, except H01 (same with EU770212.1 and EU770213.1), H02 (same with EU770207 and EU770208), H05 (same with EU770211), H06 (same with EU770215.1), H07 (same with EU770209.1 andEU770210.1), and H11 (same with EU770217.1), the remaining 22 novel *Pi-ta* haplotypes were identified compared to previous published alleles (Table S1). The *Pi-ta* site resistant to blast (base-pairs G at 2752th) was observed in 6 haplotypes, of which 5 haplotypes were from the rice landraces in Yunnan, while the remaining haplotypes were susceptible (Table S1). The variation analysis of the *Pi-ta* alleles in the CDS positions suggested that the level of variation was higher in CDS1 compared to CDS2 (Figure 1). These findings suggest that the coding sequence (CDS) of the *Pi-ta* locus exhibits nucleotide polypeptides in various rice landraces from Yunnan Province. Further, some haplotypes possess the resistance gene against *M. oryzae* that carries *AVR-Pita1*.

### 3.3. Distribution of Pi-ta Haplotype in Rice Landraces of Different Rice-Growing Regions in Yunnan

To verify the distribution of the *Pi-ta* haplotype in different rice-growing regions in Yunnan, we further analyzed 28 *Pi-ta* haplotype dates based on the location collected from the rice simples. The results indicate that the *Pi-ta* haplotypes are distributed across the rice landraces in eight distinct rice-growing regions in Yunnan (Table 4). The distribution frequency of the *Pi-ta* haplotypes was 12.2%, 0.8%, 0.3%, 0.8%, 11.9%, 19.2%, 32.2%, and 22.6% in central, eastern, northeastern, northwestern, southeastern, southern, southwestern, and western Yunnan Province, respectively. In addition, analysis of the diversity indicated that this index was 0.79, 0.67, 0, 0.67, 0.77, 0.78, 0.80, and 0.75 for central, eastern, northeastern, southeastern, southern, southwestern and western Yunnan, respectively (Table 4). In summary, the diversity index of *Pi-ta* alleles was ordered in Yunnan Province as: southwestern > central > southern > southeastern > western > eastern or northwestern > northeastern. Among 28 haplotypes of the *Pi-ta* gene, 9 haplotypes were detected in 47 rice samples from central. A total of 3 haplotypes (H06, H07, and H12 or H01, H05, and H06) were detected in 3 rice samples from eastern or northwestern, only 1 (H07) haplotype was detected in 1 rice sample from northeastern, 8 haplotypes were detected in 46 rice samples from southeastern, 12 haplotypes were detected in 74 rice samples from southern, 20 haplotypes were detected in 124 rice samples from southwestern, and 13 haplotypes were detected in 87 rice sample from western Yunnan (Table 4).

### 3.4. Variation Distributions of R/S Alleles of Pi-ta Locus Proteins in Rice Landraces in Yunnan Province

The variations of the nucleotide result in encoding the different protein products. To investigate the protein types (PT) of variants in 35 alleles of the *Pi-ta* locus, multiple amino acid alignments were performed. A total of 32 variants to amino acid sites were identified, and 22 protein products were found in the CDS regions of these *Pi-ta* loci. Of these the coding PT01, including the published haplotypes EU04, EU05, EU07, EU08, and EU09 and the haplotypes H01, H07, H09, H15, and H22 of rice landraces in Yunnan, had the highest ratio. A total of 5 *Pi-ta* haplotypes coded PT02 (EU02, EU03, H02, H10, and H20), PT04 (EU06, H04, H05, H12, and H19), and PT05(EU10, EU12, H06, H08, and H11), respectively. There were 2 *Pi-ta* coding PT16 (H25 and H28), while the remaining *Pi-ta* haplotypes coded a corresponding protein product, respectively (Table 5). Interestingly, we found 12 novel Pi-ta proteins in the 28 alleles of rice landraces in Yunnan compared to the published 16 alleles, and 5 out of 12 novel Pi-ta proteins (the haplotype H03, H13, H16, H18, and H21) were resistant to the rice blast *AVR-Pita1* gene, as the amino acid (alanine) at the position 918 was known to be a key site for resistance to rice blast [16]. A total of 62 out of 385 rice landraces in Yunnan coded to the 5 novel Pi-ta-resistant proteins (Ala-918), and the frequency was 16.1%, while the remaining rice landraces in Yunnan holding the *Pi-ta* gene were susceptible (Table 6). In summary, these results show that the *Pi-ta* gene in the rice-growing regions in Yunnan is constantly varying, and the variation of the *Pi-ta* gene population is of great significance, because in some cases, the *Pi-ta* gene has evolved haplotype alleles that resist the rice blast *AVR-Pita1* gene. Meanwhile, this evolution is enormously beneficial for the cultivation of the germplasm resources.

### 3.5. R Alleles of Pi-ta Derived from the S Alleles in Rice Landraces in Yunnan Province

**Figure 2:**
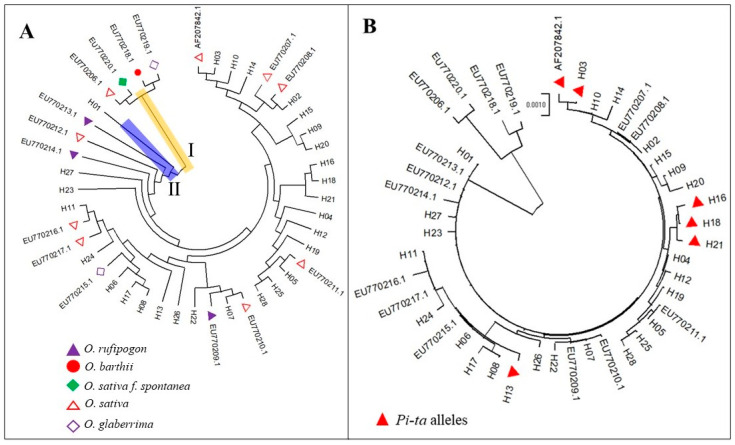
Neighbor joining phylogenetic tree of *Pi-ta* resistance (*R*)/susceptibility (*S*) alleles. (**A**), systematical evolution of 44 *Pi-ta* alleles. *R/S* alleles of the *Pi-ta* can be divided into 2 different clusters. Cluster I contained 4 *Pi-ta* haplotypes (wild *O. barthii*, *O. glaberrima*, *O. sativa f. spontanea*, and *O. sativa Indica* Group), while Cluster II contained wild *Oryza rufipogon* and *Oryza glaberrima,* and all of *Pi-ta* haplotypes in rice landraces in Yunnan. (**B**), the phylogenetic relationship of *Pi-ta R/S* alleles. The *Pi-ta R* alleles were derived from *S* alleles in rice landraces in Yunnan. These *Pi-ta* haplotype alleles were obtained from rice landraces in Yunnan (28 *Pi-ta* haplotype alleles, H01–H28) and the published GenBank (16 *Pi-ta* haplotype alleles, accession number: AF207842.1, EU770206.1, EU770207.1, EU770208.1, EU770209.1, EU770210.1, EU770211.1, EU770212.1, EU770213.1, EU770214.1, EU770215.1, EU770216.1, EU770217.1, EU770218.1, EU770219.1, EU770220.1).

To analyze the evolutionary relationships in the 28 *Pi-ta* haplotype populations and 16 *Pi-ta* variants from GenBank, a haplotype population genetic tree was constructed using the neighbor joining method. The result showed that the *Pi-ta* haplotype populations could be divided into 2 different clusters (Ⅰ and Ⅱ). Cluster Ⅰ contained 4 *Pi-ta* haplotypes (Figure 2A), including wild rice (*Oryza barthii*, EU770218.1), *Oryza glaberrima* (EU770219.1), *Oryza sativa f. spontanea*, and *Oryza sativa Indica* Group (EU770206.1), while Cluster Ⅱ contained wild rice *(Oryza rufipogon*, EU770209.1, EU770213.1, EU770214.1), *Oryza glaberrima* (EU770215.1), *Oryza sativa* (EU770217.1, EU770220.1, EU770207.1, EU770208.1, EU770210.1, EU770212.1, AF207842.1, EU770211.1, EU770216.1), and the remaining haplotypes (Figure 2A). These results show that the *Pi-ta R/S* alleles in Yunnan rice landraces were located in a same cluster of *Oryza rufipogon.* Interestingly, the *Pi-ta R* alleles of AF207842.1, H03, H13, H16, H18, and H21 were far from the *S* alleles (Figure 2B), suggesting that the *Pi-ta R* alleles were derived from *S* alleles.

### 3.6. Stepwise Evolutionary Process of Pi-ta Haplotypes in Rice Landraces in Yunnan Province

**Figure 3:**
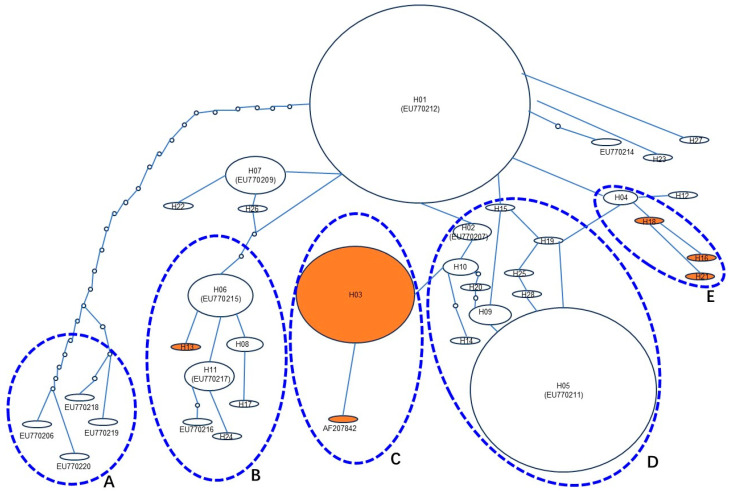
The haplotype network for the 28 *Pi-ta* alleles and the 13 reference *Pi-ta* alleles in rice. Haplotype network analysis was performed using TCS1.21 (http://darwin.uvigo.es/). The Pi-ta haplotypes were major divided into 5 evolutionary clades. Clade A contained 4 *Pi-ta* orthologues and they derived from the published sequences in GenBank. Clade D possessed the most *Pi-ta* orthologues, but not contained its *R* allele. In contrast, clade B, C, and E included the *Pi-ta R* allele at least one and derived from the *S* orthologues. The original *Pi-ta* alleles were designated as the H01 haplotype in the network. Each *Pi-ta* haplotype was separated by mutational events. The node in the network represents an extinct or a missing haplotype not found among the samples. All haplotypes were displayed as circles. The size of the circles corresponded to the haplotype frequency. H01–H28 were obtained from 385 rice landraces in Yunnan. The AF207842.1, EU770206.1, EU770207.1 (same with H02), EU770209.1 (same with H07), EU770211.1 (same with H05), EU770212.1 (same with H01), EU770214.1, EU770215.1 (same with H06), EU770216.1, EU770217.1 (same with H11), EU770218.1, EU770219.1, and EU770220.1 (GenBank accession number) of the *Pi-ta* haplotypes were obtained from GenBank. White color indicates the susceptibility alleles of *Pi-ta* gene, and yellow color indicates the resistance alleles of *Pita* gene. A to E, 5 major haplotypes of *Pi-ta* in Yunnan Province of China, are shaded.

Genetic variation is a result of long-term natural evolution. To further understand the stepwise evolutionary deductive relationship between *Pi-ta* haplotype populations, the haplotype network was developed through the TCS Network (http://darwin.uvigo.es/) based on polymorphisms nucleotide of *Pi-ta* coding for 28 alleles identified from rice landraces in Yunnan and 13 reference sequences (6 haplotype sequences from the same rice landraces in Yunnan) obtained from GenBank. A total of 5 major evolutionary clades (A, B, C, D, and E) were observed among 35 *Pi-ta* haplotype alleles (Figure 3). H01 (same with EU770212.1) was found to be the most original ancestor of the remaining *Pi-ta* haplotypes. Clade A contained 4 *Pi-ta*-susceptible orthologues (EU770206.1, EU770218.1, EU770219.1, and EU770220.1) and occurred because of the multiple mutational steps from H01. Clade B contained 7 *Pi-ta* orthologues, of which the haplotype H13 carried a resistant site (Ala-918) and evolved from a single mutational step in the *Pi-ta*-susceptible haplotype H06 (same with EU7770215.1). Clade C consisted of 2 *Pi-ta*-resistant alleles (H03 and AF207842.1). The haplotype H03 was generated from a single base mutational step in the *Pi-ta*-susceptible allelic H10. Clade D contained 12 *Pi-ta*-susceptible orthologues, while Clade E consisted of *Pi-ta R* (H16, H18, and H21) and *S* alleles (H04), in which H04 was the remaining origin of 3 *Pi-ta R* alleles. These results suggest that the B, C, and E clades of *Pi-ta R* alleles in 385 rice landraces in Yunnan evolved from *S* alleles.

### 3.7. Pi-ta Gene Undergoing the Process of Contraction in Rice Landraces in Yunnan Province

The natural selection pressure on *Pi-ta* was calculated by Tajima’s neutrality test on 385 of the *Pi-ta* CDS sequences. Tajima’s *D* value was not significantly different from 0 (*D* = 0.55445; *p* > 0.1) (Table 7). This result suggests that *Pi-ta* may suffer from balancing selection, in which the frequency of *Pi-ta R* and *S* alleles occurs at a relatively high rate in the population of 385 rice landraces in Yunnan, indicating that the *Pi-ta* populations are undergoing the process of contraction.

## 4. Discussion

Rice blast is one of the most serious diseases affecting rice crops. Disease resistance breeding is the most economically and ecological friendly way to control this disease. However, most resistance genes have a short life span in the field [9]. Therefore, it is mandatory to select germplasm resources with polygenic resistance for the sustainable control of rice blast. Currently, over 100 blast resistance genes have been identified, and among them the *Pi-ta* gene stands out as one of the most effective and durable resistance genes. In the United States, the *Pi-ta* gene has conferred resistance to the major pathogenic forms of *M*. *oryzae* [26]. A study has shown that the *Pi-ta* gene provides 14 years of durable resistance to the contemporary field populations of *M*. *oryzae* in rice-growing areas of the southern United States [27]. Some studies have reported the introduction of the *Pi-ta* gene into cultivated rice varieties from “Tetep” and “Taducan” [10,15,28]. Our results showed that approximately 95% (385 out of 405) of rice landraces collected from different regions in Yunnan Province carried the *Pi-ta* gene. These samples with the *Pi-ta* gene could be divided into 28 haplotypes based on the variation analysis of nucleotide sequences in the coding regions and found 12 novel Pi-ta protein products. The haplotype diversity of the *Pi-ta* gene may be associated with the difference in rice landraces in these different regions. Yunnan in China is known as the origin of multi-cropping agriculture, including rice [21]. This rich history and genetic diversity make it a valuable resource for ongoing research and development in disease resistance for crops such as rice. These results indicate that the haplotypes of the *Pi-ta* gene are diverse, the variation of nucleotides in its coding region is favorable, and the cultivated rice landraces in Yunnan Province have long held the *Pi-ta* gene. Thakur and his workers [20] analyzed the variation of *Pi-ta* alleles in 529 rice landraces in India and in 220 rice accessions, finding that there was a high degree of nucleotide variation in the *Pi-ta* gene in the intron region, and that 64 *Pi-ta* haplotypes and 47 Pi-ta protein variants were identified, according to the nucleotide polymorphism of the coding region and amino acid sequences of its locus, respectively. Wang et al. [13] showed that the *Pi-ta* alleles in rice accessions from 6 *Oryza spp.* (*Oryza sativa*, *Oryza glaberrima*, *Oryza rufipogon*, *Oryza nivara*, *Oryza Glaberrima*, *Oryza Rufipogon*, and *Oryza Nivara*) could be divided into 16 different haplotypes. The diversity of the *Pi-ta* gene haplotypes observed in this study aligns closely with the findings from two previously mentioned studies. The plant *R* gene polymorphism is an important part of plant innate immune resistance to pathogens, and most *R* genes are highly polymorphic and diverse [29]. Similarly, avirulence (*AVR*) genes corresponding to *R* genes are also in frequent variation. Studies have shown that the *AVR-Pita1* gene, an *AVR* gene from *M. oryzae* and the interaction with *Pi-ta* triggering a downstream immune response, is also in a condition of frequent variation [30]. Thus, the diversity of the *Pi-ta* gene haplotype in different growing regions of rice in Yunnan may also be related to the continuous variation of *AVR-Pita1*, as the host resistance gene is inclined to continuous variation when the *AVR* gene form in the pathogen is in a situation of frequent mutation [31,32]. Furthermore, previous studies have shown that the frequency of avirulent gene *AVR-Pita1* in 366 *M*. *oryzae* rice isolates from different rice regions in Yunnan fields is 46.7–72.4%, and among them, the sequenced 60 isolates code for 18 *AVR-Pita1* haplotypes, of which 6 haplotypes are virulent to the *Pi-ta R* alleles, and the mutations of *AVR-Pita1* are responsible for defeating race-specific resistance in nature [33]. Thus, the diversity of the *Pi-ta* gene haplotype in different growing regions of rice in Yunnan may also be related to the continuous variation of *AVR-Pita1*, as the host resistance gene is inclined to continuous variation when the *AVR* gene form in the pathogen is in a situation of frequent mutation [28,30,31]. This represents the coevolution of the *AVR* gene and the *R* gene with the host and pathogen interaction. In addition, the continuous variation of *AVR-Pita1* may contribute to its unique location. Specifically, *AVR-Pita1* is located on chromosome 3 of the *M. oryzae* genome and is in close proximity to a telomere [34]. The coevolution of the rice gene *Pi-ta* and *M. oryzae* gene *AVR-Pita1* is still the focus of researchers [15,16,35], probably as the pair of genes strongly supports a hypothesis for gene-to-gene. For instance, Jia et al. [36] showed that rice expressing the Pi-ta gene is resistant to isolates of *M. oryzae*, expressing *AVR-Pita1* in a gene-to-gene manner. Resistant reactions to blast were triggered by the direct interaction of *Pi-ta* with *AVR-Pita1* products, and serine instead of alanine at position 918th in the Pi-ta protein resulted in the functional loss of resistance in plants or the replacement mutation of a single amino acid at position 178th in the AVR-Pita_176_ protein also disrupted to their physical interaction in vitro. In addition, an immunoreaction mediated by *Pi-ta* was required to be assisted by the resistance gene *Ptr(t)* [18,19]. These results suggest that the resistant reaction mediated by the *Pi-ta* gene is complex in rice.

Genetic evolution might be viewed as a form of biological adaptation to environmental conditions. Lee et al.’s [35] analyses of the genetic evolution of the *Pi-ta* gene in invasive weedy rice in the United States showed that Pi-ta in weed rice in the United States can be divided into 5 clusters, containing 8 different *Pi-ta* haplotypes. Only 1 subcluster (a haplotype) in these clusters, however, contained *Pi-ta* haplotype (Ala-918) resistance to rice blast. In the current study, 35 *Pi-ta* alleles can be divided into 2 different clusters. Cluster I contained 4 *Pi-ta* haplotypes (wild *O*. *barthii*, *O*. *glaberrima*, *O*. *sativa f. spontanea*, and *O*. *sativa Indica* Group), while Cluster II contained wild *Oryza rufipogon* and *Oryza glaberrima*. Furthermore, in 28 *Pi-ta* haplotypes in rice landraces collected from different regions of Yunnan, apart from 5 haplotypes carrying *Pi-ta R* alleles, the remaining haplotypes were derived from susceptible plants holding the *Pi-ta* gene. Analysis of the molecular evolution and functional adaptability of the *Pi-ta* gene in 36 wild *Oryza rufipogon* showed that haplotype H2 (Ser-918) is the ancestor of haplotype H1 (Ala-918), and most rice accessions containing the *Pi-ta* gene belong to H2 in the 26 haplotypes; it was rare that the haplotype H1 would emerge in the process of rice cultivation and domestication and nucleotide diversity of the *Pi-ta* gene in these rice accessions [35]. Our results showed that H01 (same with EU770212.1) (Ser-918) was the ancestor of the other haplotypes. In general, the variation frequency of nucleotides in the CDS1 and CDS2 regions of the *Pi-ta* gene was low, and the results are similar to those of previous studies. To investigate the diversity of the exon and intron regions of the *Pi-ta* gene in 51 rice samples from 6 *O*. spp., the *Pi-ta* alleles were classified into 2 main branches, consisting of 16 different sequences with a large number of insertions and deletions [13]. Despite this, the DNA sequence showed 16 variations of the *Pi-ta* allele in 51 rice samples, but only 9 corresponding protein products were predicted, of which only 1 *Pi-ta* resistance allele was identified [13]. Their results suggest that the *Pi-ta* gene has a certain proportion of amino acid synonymous substitution under natural selection, and only a few of them have evolved to carry gene sites resistant to rice blast. In this study, we found 12 novel Pi-ta protein products in 385 rice landraces in Yunnan, of which 5 novel protein products (Ala-918) possessed the ability to recognize AVR-Pita1, and a total of 62 rice landraces coded these proteins. Interestingly, all of the *Pi-ta* resistance alleles in the rice landraces in Yunnan were derived from the susceptible plants carrying *Pi-ta*. Furthermore, analysis of Tajima’s neutrality test showed that the *Pi-ta* alleles could suffer from balancing selection, indicating that the *Pi-ta* locus probably maintained the diversity of the sequences via the pattern. This information indicated that the *Pi-ta* gene of rice in Yunnan is constantly subjected to variations, and the *Pi-ta* gene is always under natural selection pressure; although, it had evolved to the resistance allele. The molecular characteristics of the plant *R* gene reveal the degree of structural variation that affects its ability to detect the corresponding *AVR* genes from pathogens [37,38], and the continuous evolution of the *Pi-ta* gene further indicates that *AVR*-*Pita1* is also in a condition of constant variation. In conclusion, our findings indicate that the *Pi-ta* gene is present in rice landraces across different rice-growing regions in Yunnan. Furthermore, certain rice accessions with the *Pi-ta* gene have developed resistant alleles. This discovery significantly contributes to the rich genetic resources available for the investigation of pathogenicity and screening rice accessions for resistance to rice blast.

## 5. Conclusions

In this study, we first reported the distribution of the *Pi-ta* gene rice landraces across different rice-growing regions in Yunnan Province. We also identified the haplotypes of the *Pi-ta* gene locus within these regions and found 12 novel allelic haplotypes, including 5 sites resistant against *M. oryzae*. Initially, the rice landraces carrying the *Pi-ta* haplotypes in these regions were susceptible alleles; however, due to variations in the *Pi-ta* gene, some of these haplotypes evolved into resistant alleles against *M. oryzae* through mutation in a single amino acid at position 918.

## Figures and Tables

**Table 1:** Primer pairs used in this study.

Regions	Primers	Primer Sequences (5′→3′)	Annealing Temperature	Size (bp)
Exon1	PTA1-1F	CGAAAGAGACGAGGGATTGC	57 °C	1406
	PTA1-1R	TAGATTACCAGTATGATTCATGGCT		
Exon2	PTA2-1F	CTATTTGGCTCTAGATGATTTGGAG	59 °C	1182
	PTA2-1R	AAGAATCAGAACTCGAAGAAGCCTA		
	PTA2-2F	GTTCGCCGACTCTCGCTTC	59 °C	1144
	PTA2-2R	AAGGAGGTAAGACTAGGAACCACAC		

**Table 2:** Distributions of the CDS of the *Pi-ta* gene in rice landraces collected from different rice-growing regions in Yunnan Province.

Regions	No. of Landraces	*Pi-ta* Genotype ^a^		
		G1(+, +)	G2(−, +)	G3(+, −)
Center	48	47	1	0
Eastern	3	3	0	0
Northeastern	1	1	0	0
Northwestern	3	3	0	0
Southeastern	46	46	0	0
Southern	82	74	8	0
Southwestern	128	124	3	1
Western	94	87	6	1
Total	405	385	18	2

^a.^ The genotype G1(+, +) indicates the rice landraces containing both CDS1 and CDS2 regions of the *Pi-ta* gene. G2(−, +) indicates the rice landraces containing only CDS2 regions of the *Pi-ta* gene, and G3(+, −) indicates the rice landraces containing only CDS1 regions of the *Pi-ta* gene.

**Table 3:** Variations of the *Pi-ta* locus haplotypes in the 385 rice landraces collected from different rice-growing regions in Yunnan Province, China.

**Haplotypes**	**No.**	**Frequency** **(%)**	**Variant Locus**																
			**CDS1 Region**									**CDS2 Region**							
			17	43	108	352	385	444	474	527	807	1719	1857	1873	1922	2388	2563	2752	2766
AF207842 ^a^			T	T	G	G	G	G	C	A	G	G	T	T	T	G	A	G	A
H01	120	31.2	G	.	.	.	C	.	C	.	.	.	.	.	C	.	.	T	.
H02	6	1.6	.	.	.	.	C	.	C	.	.	.	.	.	C	.	.	T	.
H03	58	15.1	.	.	.	.	.	.	C	.	.	.	.	.	C	.	.	.	.
H04	5	1.3	G	.	.	A	C	.	C	.	.	.	.	.	C	.	.	T	.
H05	100	26	G	.	.	A	C	.	C	.	.	.	A	.	C	.	.	T	G
H06	22	5.7	G	.	.	.	C	C	.	T	.	.	.	.	C	.	.	T	.
H07	19	4.9	G	.	.	.	C	.	C	.	T	.	.	.	C	.	.	T	.
H08	6	1.6	G	.	T	.	C	C	.	T	.	.	.	.	C	.	.	T	.
H09	9	2.3	G	.	.	.	C	.	C	.	.	.	A	.	C	.	.	T	G
H10	6	1.6	.	.	.	.	.	.	C	.	.	.	.	.	C	.	.	T	.
H11	14	3.6	G	.	.	.	C	C	.	T	.	.	.	.	C	A	.	T	.
H12	1	0.3	G	.	.	A	C	.	C	.	.	.	A	.	C	.	.	T	.
H13	1	0.3	G	.	.	.	C	C	.	T	.	.	.	.	C	.	.	.	.
H14	1	0.3	.	.	.	.	.	C	C	.	.	.	.	.	C	A	.	T	.
H15	2	0.5	G	.	.	.	C	.	C	.	.	.	.	.	C	.	.	T	G
H16	1	0.3	G	G	.	A	C	.	C	.	.	.	.	.	C	.	.	.	.
H17	1	0.3	G	.	T	.	C	C	.	T	.	.	.	.	C	.	C	T	.
H18	1	0.3	G	.	.	A	C	.	C	.	.	.	.	.	C	.	.	.	.
H19	1	0.3	G	.	.	A	C	.	C	.	.	.	.	.	C	.	.	T	G
H20	2	0.5	.	.	.	.	.	.	C	.	.	.	A	.	C	.	.	T	G
H21	1	0.3	.	.	.	A	C	.	C	.	.	.	.	.	C	.	.	.	.
H22	1	0.3	G	.	.	.	C	.	C	.	T	.	A	.	C	.	.	T	.
H23	2	0.5	G	.	.	.	C	.	C	.	.	T	.	.	C	.	.	T	.
H24	1	0.3	G	.	.	.	C	C	.	T	.	T	.	.	C	A	.	T	.
H25	1	0.3	G	.	.	A	C	.	C	.	.	.	.	A	C	.	.	T	G
H26	1	0.3	G	.	.	.	C	C	C	.	T	.	.	.	C	.	.	T	.
H27	1	0.3	G	.	.	.	C	.	C	.	.	.	.	A	C	.	.	T	.
H28	1	0.3	G	.	.	A	C	.	C	.	.	.	A	A	C	.	.	T	G
Total	385	100																	

^a^ The *Pi-ta* sequence from GenBank (accession number ID: AF2027842.1).

**Table 4:** Distribution of *Pi-ta* haplotypes in different rice-growing regions in Yunnan.

Haplotypes	No.	Percent (%)	Regions							
			Centre	Eastern	Northeastern	Northwestern	Southeastern	Southern	Southwestern	Western
H01	120	31.2	10 (21.3) ^a^	0	0	1 (33.3)	8 (17.4)	26 (35.1)	38 (30.6)	37 (42.5)
H02	6	1.6	0	0	0	0	0	3 (4.1)	1 (0.8)	2 (2.3)
H03	58	15.1	6 (12.8)	0	0	0	12 (26.1)	11 (14.9)	14 (11.3)	15 (17.2)
H04	5	1.3	0	0	0	0	0	2 (2.7)	2 (1.6)	1 (1.1)
H05	100	26	14 (29.8)	1 (33.3)	0	1 (33.3)	16 (34.8)	18 (24.3)	36 (29.0)	14 (16.1)
H06	22	5.7	2 (4.3)	1 (33.3)	1 (100.0)	1 (33.3)	5 (10.9)	1 (1.4)	5 (4.0)	6 (6.9)
H07	19	4.9	0	0	0	0	0	6 (8.1)	11 (8.9)	2 (2.3)
H08	6	1.6	1 (2.1)	0	0	0	0	0	3 (2.4)	2 (2.3)
H09	9	2.3	0	0	0	0	2 (4.3)	2 (2.7)	2 (1.6)	3 (3.4)
H10	6	1.6	0	0	0	0	0	2 (2.7)	2 (1.6)	2 (2.3)
H11	14	3.6	11 (23.4)	1 (33.3)	0	0	1 (2.2)	0	1 (0.8)	0
H12	1	0.3	0	0	0	0	0	0	1 (0.8)	0
H13	1	0.3	0	0	0	0	0	0	1 (0.8)	0
H14	1	0.3	0	0	0	0	0	0	0	1 (1.1)
H15	2	0.5	0	0	0	0	1 (2.2)	0	0	1 (1.1)
H16	1	0.3	0	0	0	0	0	0	1 (0.8)	0
H17	1	0.3	1 (2.1)	0	0	0	0	0	0	0
H18	1	0.3	0	0	0	0	0	0	1 (0.8)	0
H19	1	0.3	0	0	0	0	0	1 (1.4)	0	0
H20	2	0.5	0	0	0	0	0	0	1 (0.8)	1 (1.1)
H21	1	0.3	1 (2.1)	0	0	0	0	0	0	0
H22	1	0.3	0	0	0	0	0	0	1 (0.8)	0
H23	2	0.5	0	0	0	0	1 (2.2)	0	1 (0.8)	0
H24	1	0.3	0	0	0	0	0	0	1 (0.8)	0
H25	1	0.3	0	0	0	0	0	0	1 (0.8)	0
H26	1	0.3	0	0	0	0	0	1 (1.4)	0	0
H27	1	0.3	0	0	0	0	0	1 (1.4)	0	0
H28	1	0.3	1 (2.1)	0	0	0	0	0	0	0
Total	385	100	47 (12.2) ^a^	3 (0.8)	1 (0.3)	3 (0.8)	46 (11.9)	74 (19.2)	124 (32.2)	87 (22.6)
No. of haplotypes			9	3	1	3	8	12	20	13
Index of diversity ^b^			0.79	0.67	0	0.67	0.77	0.78	0.8	0.75

^a^ Number and frequency (in bracket) of each *Pi-ta* haplotype in rice landraces in Yunnan, China. ^b^ Diversity index was calculated as the frequency of *Pi-ta* gene haplotype types in rice population following Fontaine’s method [24]. Diversity index = (1 − ∑^n^_i_ = 1*P*_i_^2^) (where *P*i is the frequency of the haplotype i in a population).

**Table 5:** Variations of the *Pi-ta* loci proteins in rice landraces in Yunnan, China.

No. ^a^	Total	Variation Positions																																
		6	15	61	79	118	148	158	162	174	176	219	230	234	315	386	395	403	416	466	479	571	573	625	641	644	711	724	816	855	887	911	918	Dis. ^b^
	AF	I	S	Y	A	G	R	H	H	E	D	V	K	I	R	V	H	M	I	H	K	A	K	C	F	L	T	R	L	T	H	P	A	R
	EU01	S	.	.	V	.	.	.	D	.	.	.	R	V	K	F	Y	I	.	R	R	S	.	.	S	I	N	C	F	.	N	L	S	S
	EU11	S	.	.	.	.	S	Q	.	.	V	A	.	.	.	.	.	.	.	.	.	.	.	.	S	.	.	.	.	.	.	.	S	S
	EU13	S	.	H	V	.	.	.	D	Q	.	.	.	V	K	.	Y	I	.	.	.	S	.	.	S	I	N	C	F	.	N	.	S	S
	EU14	S	.	H	V	.	.	.	D	.	.	.	.	V	K	.	Y	I	.	.	.	S	.	.	S	I	N	C	F	.	N	.	S	S
	EU15	S	.	.	V	.	.	.	D	.	.	.	R	V	K	F	Y	I	T	R	R	S	.	.	S	I	N	C	F	.	N	L	S	S
PT01	EU04, EU05, EU07, EU08, EU09, H01, H07, H09, H15, H22	S	.	.	.	.	.	.	.	.	.	.	.	.	.	.	.	.	.	.	.	.	.	.	S	.	.	.	.	.	.	.	S	S
PT02	EU02, EU03, H02, H10, H20	.	.	.	.	.	.	.	.	.	.	.	.	.	.	.	.	.	.	.	.	.	.	.	S	.	.	.	.	.	.	.	S	S
PT03	H03	.	.	.	.	.	.	.	.	.	.	.	.	.	.	.	.	.	.	.	.	.	.	.	S	.	.	.	.	.	.	.	.	R
PT04	EU06, H04, H05, H12, H19	S	.	.	.	S	.	.	.	.	.	.	.	.	.	.	.	.	.	.	.	.	.	.	S	.	.	.	.	.	.	.	S	S
PT05	EU10, EU12, H06, H08, H11	S	.	.	.	.	S	Q	.	.	V	.	.	.	.	.	.	.	.	.	.	.	.	.	S	.	.	.	.	.	.	.	S	S
PT06	H13	S	.	.	.	.	S	Q	.	.	V	.	.	.	.	.	.	.	.	.	.	.	.	.	S	.	.	.	.	.	.	.	.	R
PT07	H14	.	.	.	.	.	S	.	.	.	.	.	.	.	.	.	.	.	.	.	.	.	.	.	S	.	.	.	.	.	.	.	S	S
PT08	H16	S	A	.	.	S	.	.	.	.	.	.	.	.	.	.	.	.	.	.	.	.	.	.	S	.	.	.	.	.	.	.	.	R
PT09	H17	S	.	.	.	.	S	Q	.	.	V	.	.	.	.	.	.	.	.	.	.	.	.	.	S	.	.	.	.	P	.	.	S	S
PT10	H18	S	.	.	.	S	.	.	.	.	.	.	.	.	.	.	.	.	.	.	.	.	.	.	S	.	.	.	.	.	.	.	.	R
PT11	H21	.	.	.	.	S	.	.	.	.	.	.	.	.	.	.	.	.	.	.	.	.	.	.	S	.	.	.	.	.	.	.	.	R
PT12	H27	S	.	.	.	.	.	.	.	.	.	.	.	.	.	.	.	.	.	.	.	.	.	S	S	.	.	.	.	.	.	.	S	S
PT13	H23	S	.	.	.	.	.	.	.	.	.	.	.	.	.	.	.	.	.	.	.	.	N	.	S	.	.	.	.	.	.	.	S	S
PT14	H26	S	.	.	.	.	S	.	.	.	.	.	.	.	.	.	.	.	.	.	.	.	.	.	S	.	.	.	.	.	.	.	S	S
PT15	H24	S	.	.	.	.	S	Q	.	.	V	.	.	.	.	.	.	.	.	.	.	.	N	.	S	.	.	.	.	.	.	.	S	S
PT16	H25, H28	S	.	.	.	S	.	.	.	.	.	.	.	.	.	.	.	.	.	.	.	.	.	S	S	.	.	.	.	.	.	.	S	S

^a.^ Pi-ta protein numbers, including the novel types marked by the bule colors, were discovered in rice landraces in Yunnan. The AF, EU01, EU02, EU03, EU04, EU05, EU06, EU07, EU08, EU09, EU10, EU11, EU12, EU13, EU14, and EU15 haplotypes of the *Pi-ta* gene sequence are from GenBank; the accession number IDs are AF207842.1, EU770206.1, EU770207.1, EU770208.1, EU770209.1, EU770210.1, EU770211.1, EU770212.1, EU770213.1, EU770214.1, EU770215.1, EU770216.1, EU770217.1, EU770218.1, EU770219.1, and EU770220.1, respectively. The H01, H07, H09, H15, and H22 (PT01), H10 and H20 (PT02), H04, H05, H12, and H19 (PT04), and H06, H08, and H11 (PT05) were the same as the coding products that were predicted by the published sequences. ^b.^ resistant sites to rice blast disease, R, resistance (Ala-918); S, susceptibility (Ser-918) [16].

**Table 6:** Distribution of functional *Pi-ta* alleles in the 385 samples collected from different rice-growing regions in Yunnan.

	*Pi-ta* Alleles (*R*)	*Pi-ta* Alleles (*S*)	
Haplotypes	H03, H13, H16, H18, H21	H01, H02, H04, H05, H06, H07, H08, H09, H10, H11, H12, H14, H15, H17, H19, H20, H22, H23, H24, H25, H26, H27, H28	Total
No. of accessions	62	323	385
Frequency (%)	16.1	83.9	100

**Table 7:** Tajima’s neutrality test of *Pi-ta* in 385 rice landraces in Yunnan.

*N*	*S*	*k*	*π*	*D*
385	16	3.01353	0.00108	0.55445 (NS, *p* > 0.10)

The analysis involved 385 nucleotide sequences of *Pi-ta*. *N* indicates number of sequences, *S* indicates number of segregating sites, *k* indicates number of nucleotide differences, *π* indicates nucleotide diversity, and *D* is the Tajima test statistic. Tajima’s *D*: 0.55445, statistical significance: not significant, *p* > 0.10.

## Data Availability

The datasets used and/or analyzed during the current study are available from the corresponding authors on reasonable request after approval from the Agricultural Environment and Resource Research Institute, Yunnan Academy of Agricultural Sciences, in China.

## Supplementary Materials

The following supporting information can be downloaded at: https://www.mdpi.com/article/10.3390/genes15101325/s1, Table S1: Distribution of the CDS of Pi-ta gene haplotypes in the 385 rice landraces collected from the different rice-growing regions in Yunnan Province, China.

## Abbreviations

*S*: susceptibility; *R*: resistance; *AVR*: avirulence gene; *N*: number of sequences; *S*: number of segregating sites; *k*: number of nucleotide differences; *π*: indicates nucleotide diversity; *D*: the Tajima test statistic; PT: protein types; CDS: coding sequence.
